# Dynamic Recrystallization Model of High-Temperature Deformation and Finite Element Analysis of Microstructure Evolution of 14Cr1Mo Pressure Vessel Steel

**DOI:** 10.3390/ma18153531

**Published:** 2025-07-28

**Authors:** Baoning Yu, Bo Zhang, Ruxing Shi, Feng Mao, Shizhong Wei, Duhang Yang

**Affiliations:** 1 Longmen Laboratory, Luoyang 471000, China; 15038647370@163.com (R.S.); maofeng718@163.com (F.M.); hnwsz@126.com (S.W.); ydhcitic@163.com (D.Y.); 2 Luo Yang CITIC HIC Casting and Forging Co., Ltd., Luoyang 471039, China; zhangbo32103@126.com; 3 National Joint Engineering Research Center for Abrasion Control and Molding of Metal Materals, Henen University of Science and Technology, Luoyang 471000, China

**Keywords:** 14Cr1Mo, steel for pressure vessels, dynamic recrystallization, grain size, finite element simulation

## Abstract

Due to the frequent occurrence of coarse-grained structures in large hydrogenation tube sheets, their hydrogen resistance and corrosion resistance deteriorate, significantly shortening their service life. Therefore, microstructure evolution must be strictly controlled during the forging process. High-temperature compression tests were simulated using a Gleeble-1500D thermal simulator to investigate the hot deformation behavior of 14Cr1Mo pressure vessel steel under deformation conditions of 1050–1250 °C and strain rates of 0.01–1 s^−1^. Based on the experimental data, the flow stress curve of 14Cr1Mo steel was obtained, and its thermal deformation behavior was analyzed. Furthermore, the dynamic recrystallization (DRX) kinetic model and grain size model of 14Cr1Mo steel were established. These models were then integrated into the finite element software Forge^®^ to validate the accuracy of the DRX models. The results showed excellent agreement between the simulated and experimentally measured grain sizes, with a maximum deviation of less than 8%, confirming the high accuracy of the dynamic recrystallization models. These models provide a theoretical basis for finite element simulation and microstructure control in the manufacturing of super-large pressure vessel tube sheet forgings.

## 1. Introduction

With the global push toward carbon emission reduction and carbon neutrality, decarbonization efforts in high-carbon industries such as petrochemical energy have become particularly critical. Among these technologies, heavy oil conversion—which transforms highly polluting heavy oil into light oil and low-carbon olefins—plays a significant role in reducing carbon emissions [1]. As a core forged component in heavy oil conversion, the hydroprocessing reactor tube sheet serves multiple critical functions, including supporting the catalyst bed, buffering thermal stress, maintaining high-pressure sealing, and resisting sulfur and hydrogen attack [2]. Therefore, the steel used for tube sheets must exhibit high resistance to temper embrittlement, hydrogen-induced corrosion, and excellent weldability [3]. Low-alloy Cr-Mo steels are widely employed in hydroprocessing reactor tube sheet forgings due to their advantageous properties; chromium forms a dense Cr_2_O_3_ oxide film that effectively blocks hydrogen penetration, while molybdenum enhances corrosion resistance in hydrogen sulfide-containing environments. Additionally, these materials demonstrate stable mechanical properties under high temperature and pressure conditions, along with controllable heat-affected zones during welding [4].

The performance of materials is determined by their internal microstructure [5]. Consequently, extensive research has been conducted on the high-temperature microstructural evolution mechanisms of low-alloy Cr-Mo steels. Jo et al. [6] found that Cr-Mo alloys with the finest austenite grain size exhibited the highest hardness and tensile strength. Xia et al. [7] discovered that C-Mn-Mo and C-Mn-Cr steels developed fine and uniformly distributed martensite at cooling rates of 25 °C/s and 35 °C/s, respectively, resulting in optimal mechanical properties with tensile strengths of 1295 MPa and 1310 MPa. Wang et al. [8] demonstrated that grain refinement and increased chromium content could enhance tensile strength by over 200 MPa.

Dynamic recrystallization (DRX) kinetic models provide an accurate description of microstructural evolution and grain refinement. Kopp et al. [9] proposed a DRX model to represent microstructural evolution during bar rolling and performed numerical simulations. Wu et al. [10] investigated a high-strength low-alloy Ni-Cr-Mo-V steel and found that DRX occurred within the temperature range of 900–1200 °C and strain rates of 0.01–1 s^−1^, subsequently establishing a kinetic model. Mirzaee M et al. [11] conducted hot compression tests on 26NiCrMoV 14-5 steel at 850–1150 °C and strain rates of 0.001–1 s^−1^, and observed that DRX only initiated above 1000 °C. Cheng et al. [12] studied the DRX behavior of Mg-Gd-Y-Zn-Zr alloy based on the Laasraoui–Jonas model and performed finite element simulations, confirming good reliability with a maximum relative error of 12.39% between simulated and experimental values.

To precisely control the internal microstructure of tube sheet forgings in large-scale hydrogenation reactors, this study systematically investigated the high-temperature deformation behavior of 14Cr1Mo pressure vessel steel. Through Gleeble-1500D thermomechanical simulation tests, this paper obtained the flow stress curves under various processing conditions. The DRX evolution mechanism was comprehensively analyzed through detailed microstructural characterization, enabling the establishment of a robust DRX kinetics model specifically for 14Cr1Mo steel. This model was then successfully implemented in finite element software for process simulation. Through rigorous comparison between simulation results and experimental data, this paper demonstrated the high reliability of both the DRX kinetics model and numerical simulation approach, with the maximum deviation being controlled within 8%. These findings provide crucial theoretical guidance and practical tools for microstructure control in manufacturing super-large tube sheet forgings.

## 2. Materials and Methods

**Figure 1:**
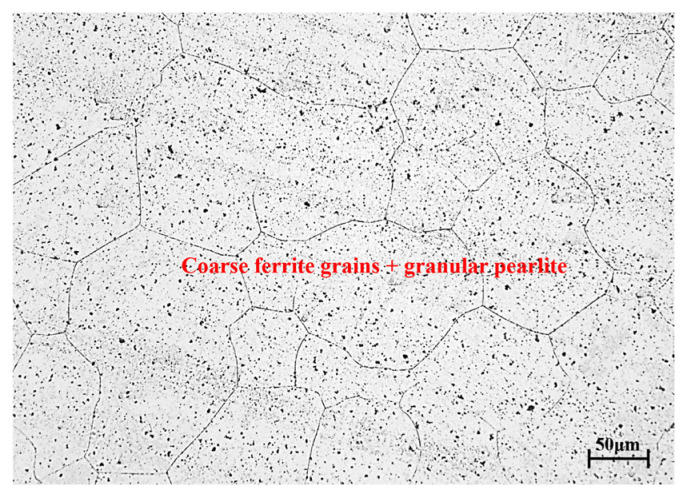
The initial microstructure of 14Cr1Mo.

**Figure 2:**
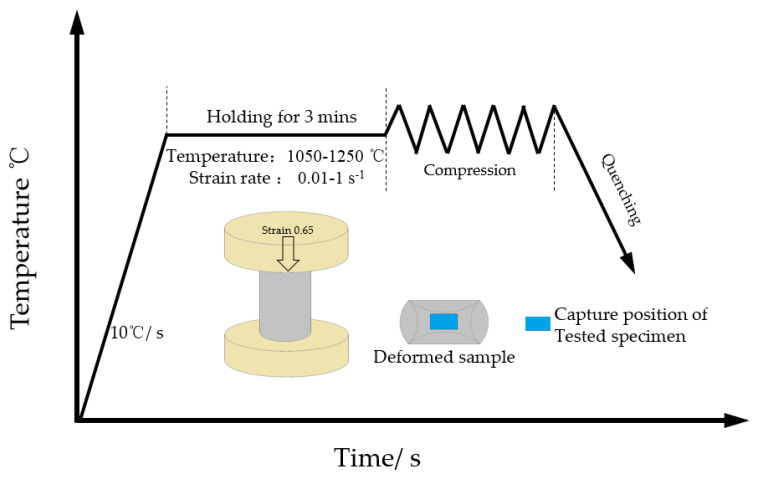
Schematic diagram of the thermal compression test of 14Cr1Mo.

For the research material, 14Cr1Mo steel was selected, with its chemical composition (mass fraction, %) presented in Table 1. The initial grain structure of 14Cr1Mo steel is shown in Figure 1; its interior was a quenched state structure, showing coarse original austenite grains and dispersed precipitated carbides. The experimental conditions included test temperatures of 1050, 1100, 1150, 1200, and 1250 °C, and strain rates of 0.01, 0.1, and 1 s^−1^, comprising a total of 21 specimens. Each cylindrical sample measured φ8×12 mm and was compressed to 50% reduction. The samples were heated to the target temperature at a constant rate of 10 °C/s, held for 3 min for temperature homogenization, and then compressed at the predetermined strain rates (as illustrated in Figure 2). Immediate water quenching was applied after compression to preserve the high-temperature microstructure. For metallographic examination, the compressed specimens were mechanically ground and polished, then etched with a nitric acid alcohol solution. Microstructural characterization was performed using optical microscopy, and the Average DRX Grain Size was quantitatively determined in accordance with the standard [13].The dynamic recrystallization behavior was systematically analyzed by correlating the microstructural observations with the corresponding stress–strain curves.

**Figure 3:**
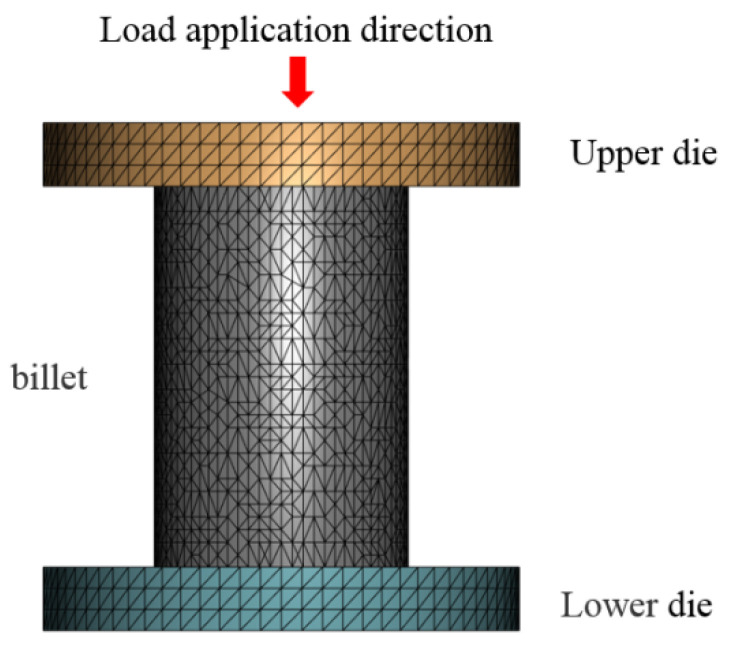
Finite element simulation model of 14Cr1Mo high-temperature compression.

A three-dimensional finite element model simulating the high-temperature compression of 14Cr1Mo was developed, as illustrated in Figure 3. The upper and lower dies measured φ12 × 3 mm, while the metal billet dimensions were φ8 × 12 mm. Using the thermomechanical simulation laboratory environment as boundary conditions, the environmental heat transfer coefficient was set to vacuum adiabatic conditions, and the friction coefficient between the dies and billet was assigned a value of 0.3.

## 3. Results

### 3.1. The Flow Stress Curve of 14Cr1Mo Steel

**Figure 4:**
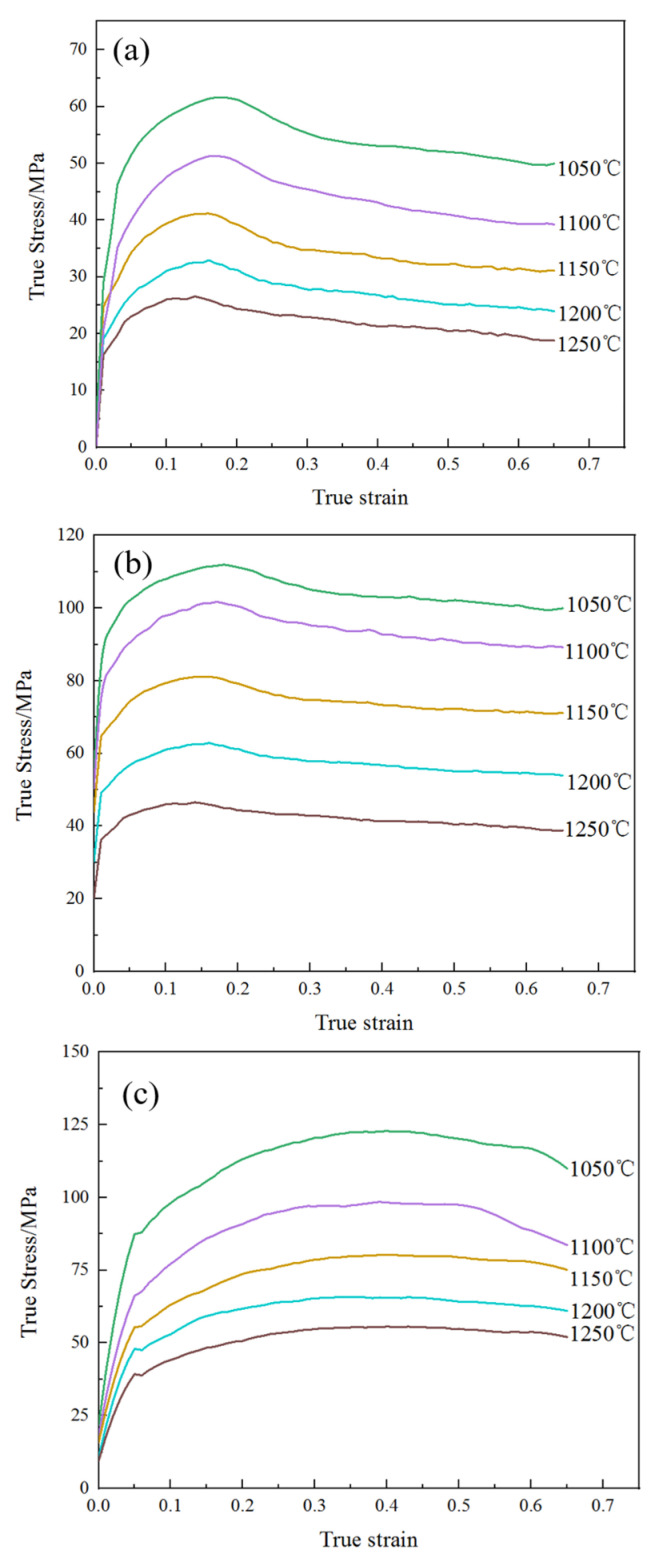
True stress–strain curve of 14Cr1Mo steel: (**a**) 0.01 s^−1^, (**b**) 0.1 s^−1^, (**c**) 1 s^−1^.

The true stress–strain curves of 14Cr1Mo steel are presented in Figure 4. As illustrated, the flow stress curves exhibited typical dynamic recrystallization (DRX) characteristics. During the initial compression stage, the stress rapidly increased due to dislocation accumulation [14,15,16]. Upon reaching the critical stress, new grains began to form through DRX, causing the stress growth rate to decelerate. In this phase, work hardening dominated, driving the stress to its peak value. Subsequently, during dynamic softening, dynamic recovery (DRV) and DRX became predominant as new grains nucleated and grew [17,18,19,20]. Finally, in the steady-state stage, the stress gradually decreased with increasing strain until equilibrium was achieved between DRV, DRX, and work hardening [21].

As shown in Figure 4, when the strain rate was constant, the decrease in deformation temperature gradually increased the peak strain of 14Cr1Mo steel. For instance, at a strain rate of 0.1 s^−1^, the temperature dropped from 1250 °C to 1050 °C, and the peak strain increased from 0.139 to 0.187 (Table 2). This was because the dynamic recrystallization of the material required sufficient activation energy. The decrease in deformation temperature made the material need a greater degree of strain, increasing the dislocation density to enhance the driving force it requires.

When the deformation temperature was constant, at low strain rates (0.01 s^−1^ and 0.1 s^−1^), the true stress of 14Cr1Mo steel tended to level off when the strain reached 0.65, indicating that the internal dynamic recrystallization of its microstructure was sufficient. At high strain rates (1 s^−1^), after the deformation of the material ends, the true stress still showed a clear tendency to decrease and did not tend to level off. Among them, this was most evident under the condition of 1050 °C/1 s^−1^. This was because the strain rate was too fast, the deformation time was too short, and the dynamic recrystallization could not occur completely in time. The degree of internal recrystallization was lower compared to the strain rates of 0.01 s^−1^ and 0.1 s^−1^.

**Figure 5:**
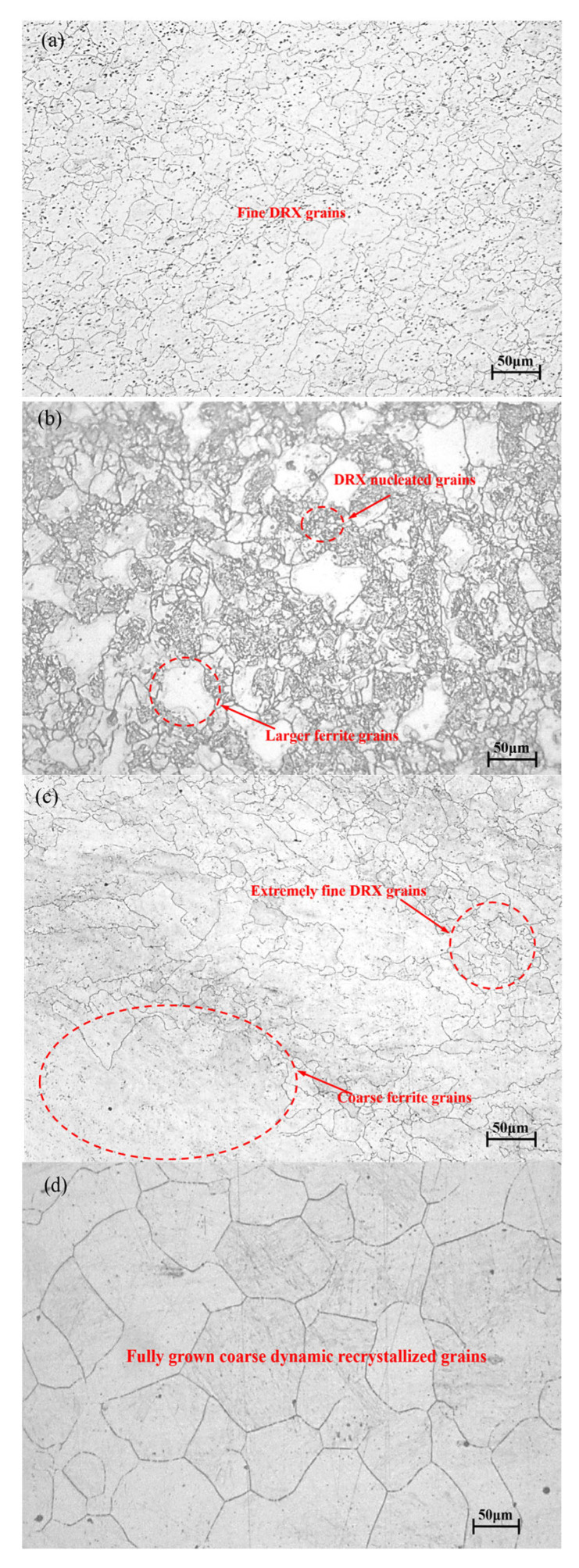
Microstructure image of 14Cr1Mo steel: (**a**) 1050 °C/0.01 s^−1^, (**b**) 1050 °C/0.1 s^−1^, (**c**) 1050 °C/1 s^−1^, (**d**) 1250 °C/0.01 s^−1^.

Under the condition of 1050 °C/0.01 s^−1^, the material reached its peak stress of 61.79 MPa at a strain of 0.175(Table 2). With further deformation, DRV and DRX continuously occurred, leading to stress reduction until a steady-state stress of 49.63 MPa was attained. The corresponding microstructure (Figure 5a) demonstrated complete DRX, featuring fine and uniform grains with an average size of approximately 34.75 μm, along with minor dispersed precipitates. However, when the strain rate remains constant and the temperature is raised to 1250 °C, although dynamic recrystallization has already occurred, the already refined grains grow again, as shown in Figure 5d, with a grain size of 136.57 μm.

When the strain rate was increased to 0.1 s^−1^, the material reached the peak stress of 114.27 MPa at a strain of 0.187 (Table 2). With the increase of strain, the stress decreased and reached a steady state of 103.79 MPa. The corresponding microstructure (Figure 5b) showed a large number of dynamic recrystallized nucleated grains and some relatively coarse grains, with an average grain size of approximately 13.32 μm.

When the strain rate was increased to 1 s^−1^, the stress initially rose sharply with increasing strain before slowing due to DRV-induced softening. The peak stress of 122.69 MPa occurred at a strain of 0.32, followed by a gradual decline to 111.80 MPa at a strain of 0.65. The absence of stress stabilization indicated incomplete DRX. The corresponding microstructure (Figure 5c) showed that the material simultaneously presented fine ferrite grains and relatively coarse ferrite grains, with poor uniformity and no obvious granular pearlite (carbides) [22]. The refined grain size measured approximately 8.16 μm.

### 3.2. Establishment of the Recrystallization Model of 14Cr1Mo Steel

#### 3.2.1. Identification Parameters of the Critical Strain Model of 14Cr1Mo Steel

Dynamic recrystallization of 14Cr1Mo steel was the process of forming new equiaxed crystals within the material. When the critical strain was reached, dynamic recrystallization could occur [23]. When the material underwent recrystallization, there was an inflection point for the work hardening rate [24]. The value was obtained through Equation (1) [25]:

(1) θ=∂σ/∂ε

Among them, σ is stress, θ is work hardening rate, and ε is strain.

Therefore, the following equation was derived:

(2) $$\frac{\partial \theta }{\partial \sigma }=\frac{\partial \theta }{\partial \varepsilon }\times \frac{\partial \varepsilon }{\partial \sigma }=\frac{\partial \theta }{\partial \varepsilon }\times \frac{1}{\theta }=\frac{\partial (\ln\theta )\theta }{\partial \varepsilon }$$

The critical strain value was determined by identifying the extremum point of curve ∂(lnθ)θ/∂ε−ε. The correlation between parameters lnθ and ε was effectively characterized by a third-order polynomial regression model, as expressed below:

(3) $$\ln\theta =A+B\varepsilon +C\varepsilon^{2}+D\varepsilon^{3}$$

Furthermore, the critical strain was obtained through Equation (4):

(4) $$\varepsilon_{c}=-\frac{C}{3D}$$

**Figure 6:**
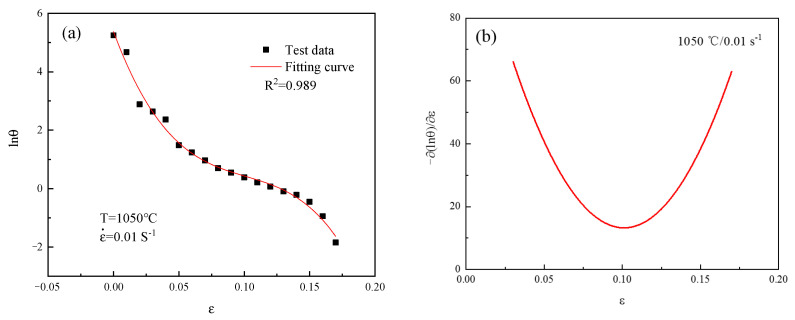
Critical strain data fitting curve graph: (**a**) lnθ−ε; (**b**)  −∂(ln(θ))/∂ε−ε.

The test data of 14Cr1Mo steel were fitted. Among them, at 1050 °C/0.01 s^−1^, lnθ−ε curve is shown in Figure 6a. This behavior was mathematically described by the following fitted curve:

(5) $$\ln\theta =7.912-43.11\varepsilon +200.003\varepsilon^{2}-375.577\varepsilon^{3}$$

Figure 6b shows the curve of  −∂(ln(θ))/∂ε−ε fitting under the condition of 1050 °C/0.01 s^−1^. The minimum point of this curve corresponded to the critical strain for 14Cr1Mo steel, with the quantitative values being summarized in Table 3.

The relationship between critical strain and peak strain was expressed as

(6) $$\varepsilon_{c}=a\cdot \varepsilon_{p}$$

The peak strain model of 14Cr1Mo steel was expressed as follows [26]:

(7) $$\varepsilon_{p}=A_{P}\cdot {\dot{\varepsilon }}^{m_{p}}\cdot \exp(\frac{Q_{P}}{RT})$$

In the formula, $\varepsilon_{p}$ is the peak strain, $\dot{\varepsilon }$ is the strain rate, $Q_{P}$ is the activation energy, T is the temperature, and the rest are constants.

**Figure 7:**
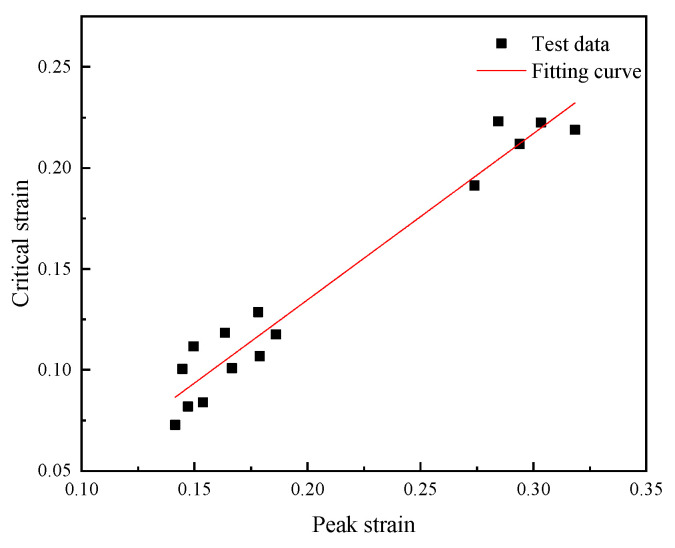
Fitting curves of $\varepsilon_{p}-\varepsilon_{c}$ at different temperatures and strain rates.

As shown in Figure 7, the peak strain and critical strain of 14Cr1Mo steel under various deformation conditions were linearly fitted to obtain slope of a=0.824, and the correlation coefficient was $R^{2}=0.977$.

**Figure 8:**
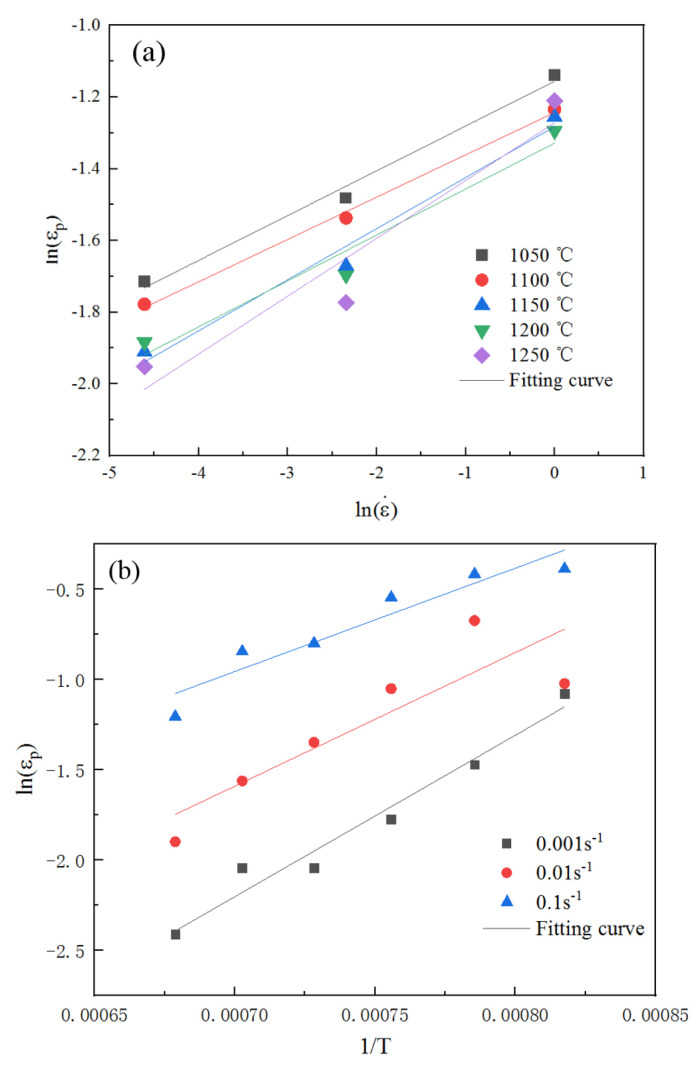
Peak strain model data fitting curve. (**a**) $\ln(\varepsilon_{p})-\ln(\dot{\varepsilon })$ fitting curves at different temperatures. (**b**) $\ln\varepsilon_{p}-1/T$ fitting curve under different strain rates.

A linear analysis of $\ln(\varepsilon_{p})-\ln(\dot{\varepsilon })$ was performed, as illustrated in Figure 8a, where the slope corresponded to parameter $m_{p}$. The average value of $m_{p}$ at different temperatures was determined to be 0.135.

The slope of $\ln\varepsilon_{p}-1/T$ was found to be $Q_{P}/R$ through linear analysis, as shown in Figure 8b. The average value of $Q_{P}/R$ across different temperatures was determined to be 1338.790 J/mol, so $Q_{P}=11130.704$ J/mol.

By substituting $m_{p}$ and $Q_{P}$ into Equation (8), the value of $A_{P}$ under each deformation condition was calculated, and the arithmetic mean value was determined to be 1.423.

Then, the critical strain calculation formula was expressed as follows:

(8) $$\varepsilon_{c}=0.824\varepsilon_{p}$$

(9) $$\varepsilon_{p}=1.423{\dot{\varepsilon }}^{0.135}\cdot \exp(\frac{11130.704}{RT})$$

#### 3.2.2. Determination of Parameters of Dynamic Recrystallization Model of 14Cr1Mo Steel

The commonly used methods for determining the volume fraction of dynamic recrystallization (DRX) in materials included metallographic analysis, the energy method, and true stress–strain curve analysis [27]. However, the metallographic approach imposed stringent requirements on specimen preparation and etching results, while the energy method proved inconvenient for measuring deformation energy storage. Consequently, the true stress–strain curve method was selected to quantify the DRX volume fraction in 14Cr1Mo steel.

The relationship between the percentage of dynamic recrystallization and stress was expressed as follows [28]:

(10) $$X_{drx}=\frac{\sigma_{drv}-\sigma }{\sigma_{sat}-\sigma_{ss}}$$

Among them, $\sigma_{drv}$ represents the flow stress value considering only dynamic recovery (DRV), the stress value obtained from the dynamic recovery curve of the material; $\sigma_{sat}$ represents the saturation stress of the DRV curve; and $\sigma_{ss}$ represents steady-state stress. Poliak [29] discovered that $\sigma_{sat}$ can be obtained through the θ−σ curve, as shown in Figure 8a, and its inflection point was the critical stress point. Furthermore, $\sigma_{drv}$ was obtained by solving Equation (13):

(11) $$\sigma_{drv}=\sigma_{sat}+(\sigma_{c}-\sigma_{sat})\exp(\frac{(\varepsilon -\varepsilon_{c})\theta_{c}}{\sigma_{c}-\sigma_{sat}})$$

**Figure 9:**
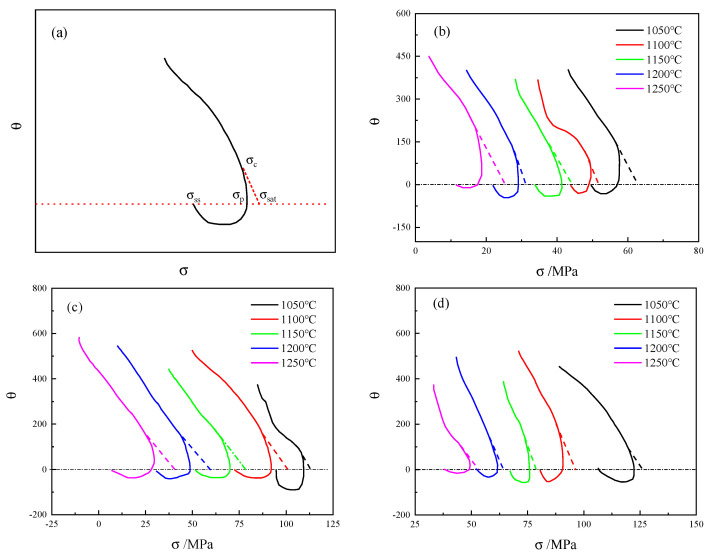
(**a**) The typical curve; (**b**) 0.01 s^−1^; (**c**) 0.1 s^−1^; (**d**) 1 s^−1^. The dotted line represents the tangent line drawn from $\sigma_{c}$ to determine the value of $\sigma_{sat}$, as shown in (**a**).

The curves of 14Cr1Mo steel under the conditions of 0.01 to 1 s^−1^ are shown in Figure 9b–d, and the obtained $\sigma_{sat}$ data are presented in Table 4.

**Figure 10:**
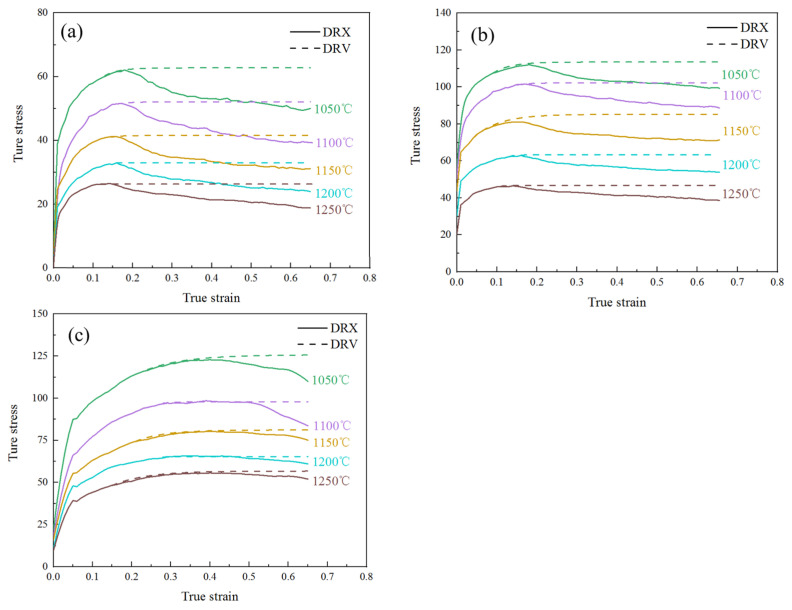
Saturated stress curves of 14Cr1Mo steel at different strain rates: (**a**) 0.01 s^−1^, (**b**) 0.1 s^−1^, (**c**) 1 s^−1^.

According to Formula (13), the saturated stress curves of 14Cr1Mo steel under different conditions were drawn as shown in Figure 10.

**Figure 11:**
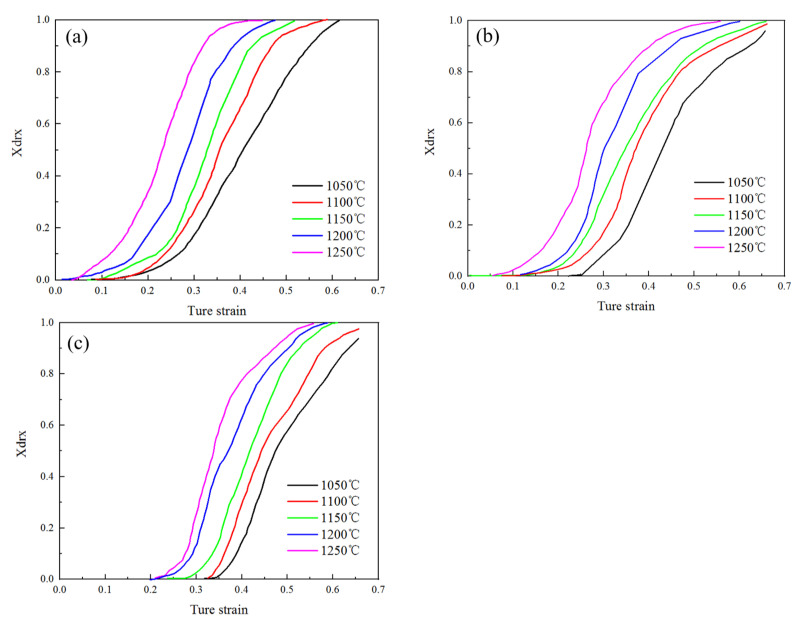
Dynamic recrystallization percentage of 14Cr1Mo steel under different deformation conditions: (**a**) 0.01 s^−1^, (**b**) 0.1 s^−1^, (**c**) 1 s^−1^.

According to Formula (12), the dynamic recrystallization percentage of 14Cr1Mo steel under different conditions was calculated, and the relationship between strain and dynamic recrystallization percentage and dynamic recrystallization characteristic strain $\varepsilon_{0.5}$ were obtained, as shown in Figure 11 and Table 5.

The dynamic recrystallization kinetic equation proposed by Kopp et al. was expressed by the following formula [9]:

(12) $$\left\{\begin{array}{l} X_{drx}=1-\exp\left[-k_{d}{\left(\frac{\varepsilon -\varepsilon_{c}}{\varepsilon_{0.5}-\varepsilon_{c}}\right)}^{n_{drx}}\right]\\ \varepsilon_{0.5}=A_{0.5}{\dot{\varepsilon }}^{m_{0.5}}\cdot \exp(\frac{Q_{0.5}}{RT}) \end{array}\right.$$

Among them, *X*_drx_ represents the volume fraction of dynamic recrystallization, ε_0.5_ represents the characteristic strain of 14Cr1Mo steel under different conditions, ε_c_ represents the strain of critical 14Cr1Mo steel under different conditions, and *k*_d_, *n*_drx_, $A_{0.5}$, $m_{0.5}$, and $Q_{0.5}$ are constants.

The expression of characteristic strain was similar to that of peak strain Formula (8). So, *m*_0.5_ = 0.0612; *Q*_0.5_ = 24,466.663 J/mol; and *A*_0.5_ = 0.0478 were calculated by the same method. Then, the characteristic strain model of 14Cr1Mo steel was obtained:

(13) $$\varepsilon_{0.5}=0.0478{\dot{\varepsilon }}^{0.0612}\cdot \exp(\frac{24466.766}{RT})$$

where ε = ε_0.5_; *X*_drx_ = 0.5; and *k*_d_ = ln2 = 0.693.

**Figure 12:**
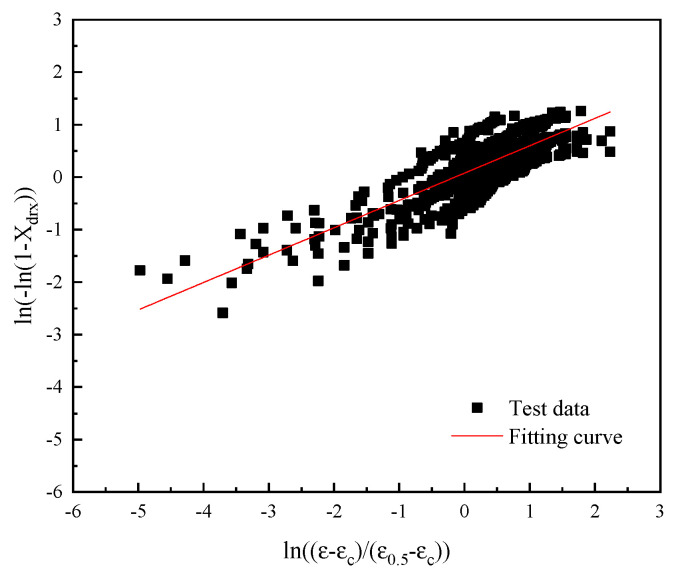
The fitting curves of $\ln[-\ln(1-X_{drx})]$ and $\ln[(\varepsilon -\varepsilon_{c})/(\varepsilon_{0.5}-\varepsilon_{c})]$.

A linear fitting was performed on $\ln[-\ln(1-X_{drx})]$ and $\ln[(\varepsilon -\varepsilon_{c})/(\varepsilon_{0.5}-\varepsilon_{c})]$, and its slope was the value of $n_{drx}$, so $n_{drx}=0.522$, as shown in Figure 12.

Then the dynamic recrystallization percentage model of 14Cr1Mo steel was

(14) $$\left\{\begin{array}{l} X_{drx}=1-\exp\left[-0.693{\left(\frac{\varepsilon -\varepsilon_{c}}{\varepsilon_{0.5}-\varepsilon_{c}}\right)}^{0.522}\right]\\ \varepsilon_{0.5}=0.0478{\dot{\varepsilon }}^{0.0612}\cdot \exp(\frac{24466.766}{RT}) \end{array}\right.$$

#### 3.2.3. Establishment of the Dynamic Recrystallization Grain Size Model

The hot compression specimens of 14Cr1Mo steel were sectioned axially, mounted, etched with an aqueous solution of ferric chloride and hydrochloric acid, and subsequently examined for metallographic characterization. The resulting grain sizes measured under various deformation conditions are presented in Table 6.

**Figure 13:**
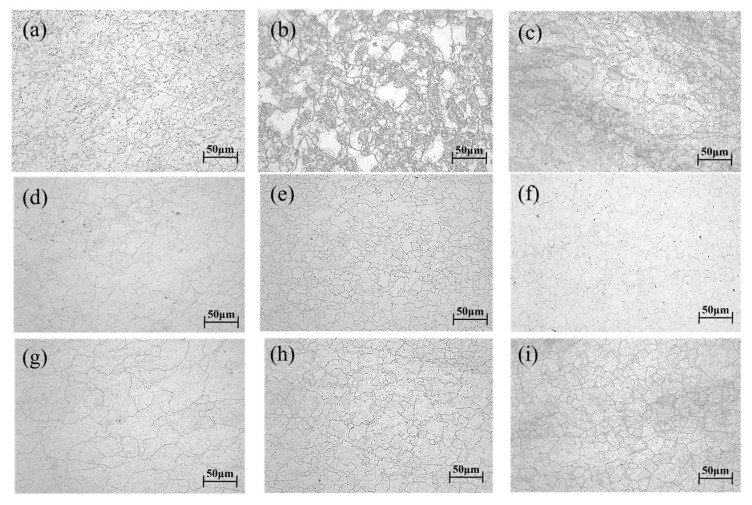

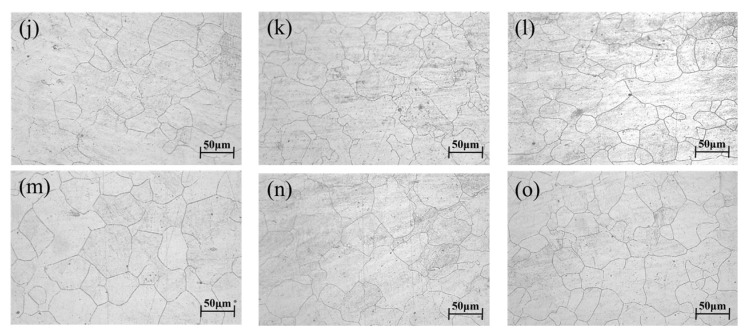
Microstructure of 14Cr1Mo steel under different conditions. (**a**) 1050 °C/0.01 s^−1^; (**b**) 1050 °C/0.1 s^−1^; (**c**) 1050 °C/1 s^−1^; (**d**) 1100 °C/0.01 s^−1^; (**e**) 1100 °C/0.1 s^−1^; (**f**) 1100 °C/1 s^−1^; (**g**) 1150 °C/0.01 s^−1^; (**h**) 1150 °C/0.1 s^−1^; (**i**) 1150 °C/1 s^−1^; (**j**) 1200 °C/0.01 s^−1^; (**k**) 1200 °C/0.1 s^−1^; (**l**) 1200 °C/1 s^−1^; (**m**) 1250 °C/0.01 s^−1^; (**n**) 1250 °C/0.1 s^−1^; (**o**) 1250 °C/1 s^−1^.

Analysis of Figure 13 revealed that at lower temperatures (1050 °C) with strain rates exceeding 0.1 s^−1^, dynamic recrystallization (DRX) did not reach completion (<100%). Incomplete DRX manifested as fine recrystallized grains along grain boundaries coexisting with relatively coarse original grains. As temperature increased, even at high strain rates, DRX progression became more substantial, resulting in significantly improved grain uniformity [30].

The classic formula of the dynamic recrystallization grain size model was as follows:

(15) $$D_{drx}=A_{D}{\dot{\varepsilon }}^{m_{D}}\cdot \exp(\frac{Q_{D}}{RT})$$

**Figure 14:**
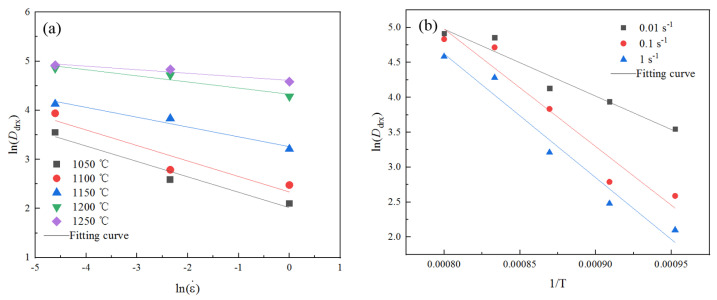
Data fitting curve of dynamic recrystallization grain size model. (**a**) $\ln(D_{drx})-\ln\dot{\varepsilon }$ fitting curve graphs at different temperatures. (**b**) $\ln(D_{drx})-1/T$ fitting curve graphs at different strain rates.

A linear analysis of $\ln(D_{drx})-\ln\dot{\varepsilon }$ was performed, as illustrated in Figure 14a, where the slope corresponded to parameter $m_{D}$. The average value of $m_{D}$ at different temperatures was determined to be −0.205.

The slope of $\ln(D_{drx})-1/T$ was $Q_{D}/R$ through linear analysis, as shown in Figure 14b. The average value of $Q_{P}/R$ at different temperatures was calculated, which was −14,719.637 J/mol. Then, $Q_{D}=-122,379.061$ J/mol.

Parameters $m_{D}$ and $Q_{D}$ were substituted into Equation (15), and the $A_{D}$ values were calculated for each deformation condition. The arithmetic mean of $A_{D}$ was determined to be 81,957.928.

Therefore, the expression for the grain size of dynamic recrystallization of 14Cr1Mo steel was

(16) $$D_{drx}=81957.928{\dot{\varepsilon }}^{-0.205}\cdot \exp(\frac{-122379.061}{RT})$$

#### 3.2.4. Verification of the Dynamic Recrystallization Kinetic Model of 14Cr1Mo Based on Finite Element Simulation

Reliable finite element simulation results depended on an accurate recrystallization kinetics model [31,32,33]. To validate the recrystallization kinetics model for 14Cr1Mo steel, the established dynamic recrystallization models (Equations (10), (11), (18), and (20)) were implemented in the finite element software Forge^®^ NxT 3.2.

**Figure 15:**
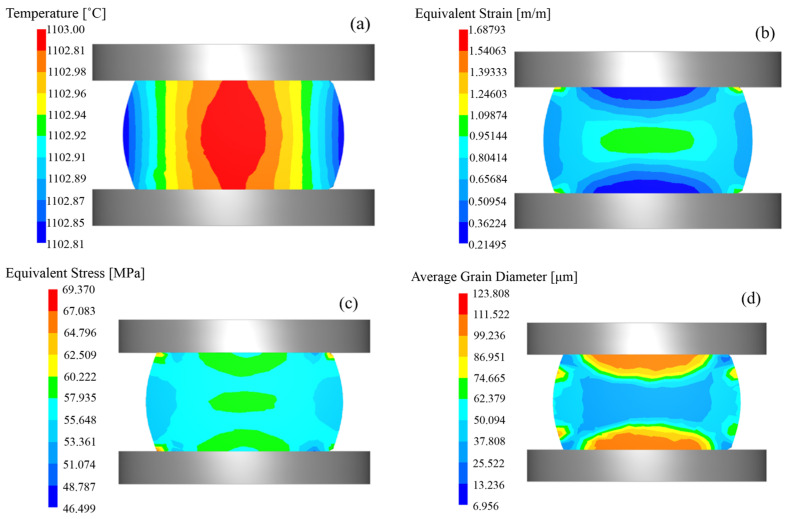
Finite element simulation cloud image of 14Cr1Mo high-temperature compression at 1100 °C/0.01 s^−1^. (**a**) Temperature field. (**b**) Strain field. (**c**) Stress field. (**d**) Grain size.

Figure 15 presents the finite element simulation results of thermomechanical compression under the deformation condition of 1100 °C/0.01 s^−1^. The results revealed that specimen deformation generated significant deformation heat, causing the specimen temperature to exceed the set temperature by 2–3 °C upon completion of compression. The central region exhibited strain values exceeding 0.95 and stress levels above 57.93 MPa, with a corresponding grain size of 50.09 μm.

**Figure 16:**
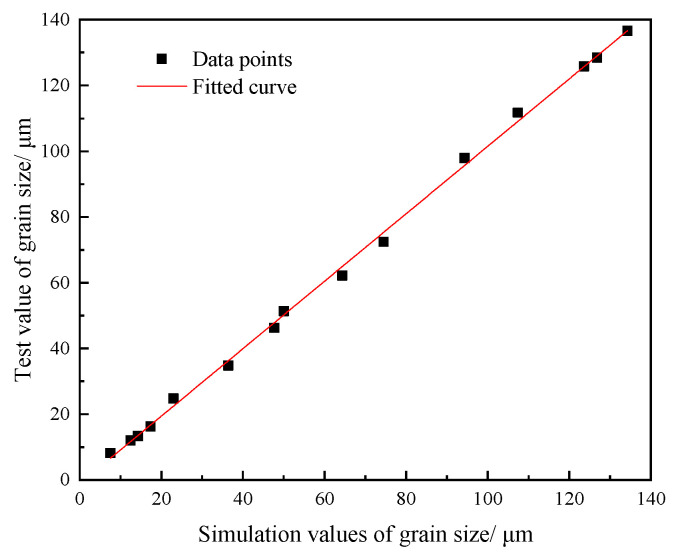
Comparison of grain sizes between finite element simulation and test value.

Finite element simulations were performed on fifteen sets of thermomechanically compressed specimens. The simulated dynamically recrystallized grain sizes are presented in Table 7. Deviation analysis was conducted by comparing simulated results with experimentally measured grain sizes. The maximum deviation (7.59%) occurred under the 1050 °C/1 s^−1^ deformation condition, while the minimum deviation (1.26%) was observed at 1200 °C/0.01 s^−1^. Figure 16 showed the comparison of the grain size of 14Cr1Mo in the finite element simulation with that test data. The correlation coefficient R was 0.998, indicating a high degree of agreement between the two. These results demonstrated the accuracy of the 14Cr1Mo recrystallization kinetics model.

## 4. Conclusions

To effectively predict and control microstructure and grain size evolution in tube sheets, this study systematically investigated the dynamic recrystallization (DRX) behavior and microstructural evolution of 14Cr1Mo steel during hot-forming processes through integrated DRX modeling and finite element simulation. The research establishes a reliable theoretical framework for optimizing hot working parameters and manufacturing ultra-large tube sheets with enhanced microstructural properties. The principal findings are summarized as follows.

High-temperature compression tests were performed on 14Cr1Mo steel using a Gleeble-1500D thermomechanical simulator (Poestenkill, NY, USA) across temperatures of 1050–1200 °C and strain rates of 0.01–1 s^−1^. The acquired true stress–strain curves displayed characteristic DRX behavior, featuring initial stress escalation to a peak value followed by gradual decline and stabilization.

Building upon experimental data and the Kopp DRX framework, a comprehensive DRX model for 14Cr1Mo steel was developed:

$$\left\{\begin{array}{l} X_{drx}=1-\exp\left[-0.693{\left(\frac{\varepsilon -\varepsilon_{c}}{\varepsilon_{0.5}-\varepsilon_{c}}\right)}^{0.522}\right]\\ \varepsilon_{0.5}=0.0478{\dot{\varepsilon }}^{0.0612}\cdot \exp(\frac{24466.766}{RT})\\ D_{drx}=81957.928{\dot{\varepsilon }}^{-0.205}\cdot \exp(\frac{-122379.061}{RT}) \end{array}\right.$$

Model validation was achieved through implementation in Forge^®^ finite element software via secondary development. Simulation of all 15 hot compression tests under identical experimental conditions yielded strong agreement with experimental measurements, demonstrating maximum grain size deviations below 8%. This confirms the model’s predictive accuracy for industrial applications.

The established model provides critical theoretical guidance for forging process optimization and grain refinement control in tube sheet manufacturing, particularly for ultra-large components requiring precise microstructure engineering.

## Figures and Tables

**Table 1:** Chemical composition of 14Cr1Mo steel (mass fraction, %).

Chemical Composition	C	Si	Mn	S	P	Cr	Ni	Mo	Cu	O	N	Ti
14Cr1Mo	0.14	0.51	0.70	0.002	0.006	1.45	0.17	0.60	0.04	0.0014	0.009	0.008

**Table 2:** Peak true stress and steady-state true stress of 14Cr1Mo under different conditions.

Temperature/°C	Strain Rates/s^−1^	Peak True Strain	Peak True Stress/MPa	Steady State True Strain	Steady State True Stress/Mpa
1050	0.01	0.175	61.79	0.616	49.63
1050	0.1	0.187	114.27	0.623	103.79
1050	1	0.32	122.69	-	-
1100	0.01	0.169	51.81	0.597	39.02
1100	0.1	0.176	101.05	0.614	88.46
1100	1	0.291	97.81	-	-
1150	0.01	0.148	41.13	0.521	31.03
1150	0.1	0.154	81.21	0.58	71.11
1150	1	0.285	82.1	-	-
1200	0.01	0.152	32.8	0.473	23.83
1200	0.1	0.15	62.16	0.51	53.48
1200	1	0.274	66.29	-	-
1250	0.01	0.142	26.07	0.442	18.88
1250	0.1	0.139	46.51	0.49	38.11
1250	1	0.298	55.81	-	-

Note: “-” indicates that there is no target parameter under this condition.

**Table 3:** Critical strain and peak strain of 14Cr1Mo steel under different deformation conditions.

Temperature/°C	Strain Rate/s^−1^	Fitting Parameters					Critical Strain	Peak Strain
		A	B	C	D	R^2^		
1050	0.01	5.374	−120.441	1059.767	−3493.4	0.989	0.101121	0.175
	0.1	4.87	−99.435	1050.002	−4271.89	0.97	0.081931	0.187
	1	4.801	−64.657	291.4233	−460.18	0.96	0.211094	0.32
1100	0.01	5.992	−138.653	1354.864	−4838.21	0.977	0.093345	0.169
	0.1	5.276	−100.069	726.491	−1804.29	0.977	0.134215	0.176
	1	4.186	−42.286	140.29	−195.433	0.94	0.239281	0.291
1150	0.01	5.1	−111.622	1163.015	−4930.67	0.985	0.078625	0.148
	0.1	4.992	−87.704	607.913	−1536.53	0.985	0.13188	0.154
	1	4.496	−54.5108	224.687	−337.614	0.97	0.221838	0.285
1200	0.01	5.996	−154.922	1799.16	−7604.61	0.97	0.078863	0.152
	0.1	4.819	−89.508	671.526	−1909.39	0.97	0.117232	0.15
	1	3.897	−46.2812	200.976	−350.378	0.96	0.191199	0.274
1250	0.01	6.117	−171.258	2255.734	−11148.4	0.98	0.067445	0.142
	0.1	4.507	−85.406	709.72	−2173.22	0.98	0.108858	0.139
	1	3.668	−39.845	158.447	−258.86	0.96	0.204032	0.298

**Table 4:** Saturated steady state under different deformation conditions.

Steady Stress/MPa	1050 °C	1100 °C	1150 °C	1200 °C	1250 °C
0.01 s^−1^	63.348	52.101	43.619	30.134	27.509
0.1 s^−1^	114.158	101.242	85.281	63.341	48.753
1 s^−1^	125.78	96.943	79.159	64.388	54.796

**Table 5:** Characteristic strain under different deformation conditions.

Characteristic Strain	1050 °C	1100 °C	1150 °C	1200 °C	1250 °C
0.01 s^−1^	0.39	0.35	0.32	0.27	0.23
0.1 s^−1^	0.42	0.37	0.34	0.29	0.26
1 s^−1^	0.48	0.44	0.42	0.37	0.34

**Table 6:** Dynamic recrystallization grain size under different deformation conditions (unit: μm).

	0.01 s^−1^	0.1 s^−1^	1 s^−1^
1050 °C	34.75	13.32	8.16
1100 °C	51.36	16.27	11.93
1150 °C	62.16	46.25	24.78
1200 °C	128.43	111.73	72.41
1250 °C	136.57	125.76	97.91

**Table 7:** Dynamic recrystallization grain size (unit: μm) simulated by simulation.

	0.01 s^−1^	0.1 s^−1^	1 s^−1^
1050 °C	36.43	14.27	7.54
1100 °C	50.09	17.32	12.53
1150 °C	64.37	47.68	22.94
1200 °C	126.81	107.43	74.52
1250 °C	134.28	123.58	94.32

## Data Availability

The original contributions presented in this study are included in the article material. Further inquiries can be directed to the corresponding author.
